# IDH1 mutation inhibits differentiation of astrocytes and glioma cells with low oxoglutarate dehydrogenase expression by disturbing α-ketoglutarate-related metabolism and epigenetic modification

**DOI:** 10.1093/lifemeta/loae002

**Published:** 2024-01-15

**Authors:** Yuanlin Zhao, Ying Yang, Risheng Yang, Chao Sun, Xing Gao, Xiwen Gu, Yuan Yuan, Yating Nie, Shenhui Xu, Ruili Han, Lijun Zhang, Jing Li, Peizhen Hu, Yingmei Wang, Huangtao Chen, Xiangmei Cao, Jing Wu, Zhe Wang, Yu Gu, Jing Ye

**Affiliations:** State Key Laboratory of Holistic Integrative Management of Gastrointestinal Cancers, Department of Pathology, Xijing Hospital and School of Basic Medicine, Fourth Military Medical University, Xi’an, Shaanxi 710032, China; State Key Laboratory of Holistic Integrative Management of Gastrointestinal Cancers, Department of Pathology, Xijing Hospital and School of Basic Medicine, Fourth Military Medical University, Xi’an, Shaanxi 710032, China; State Key Laboratory of Holistic Integrative Management of Gastrointestinal Cancers, Department of Pathology, Xijing Hospital and School of Basic Medicine, Fourth Military Medical University, Xi’an, Shaanxi 710032, China; Department of Pathology, Air Force Hospital of Southern Theater Command, Guangzhou, Guangdong 510000, China; Department of Neurology, Tangdu Hospital, Fourth Military Medical University, Xi’an, Shaanxi 710038, China; State Key Laboratory of Holistic Integrative Management of Gastrointestinal Cancers, Department of Pathology, Xijing Hospital and School of Basic Medicine, Fourth Military Medical University, Xi’an, Shaanxi 710032, China; State Key Laboratory of Holistic Integrative Management of Gastrointestinal Cancers, Department of Pathology, Xijing Hospital and School of Basic Medicine, Fourth Military Medical University, Xi’an, Shaanxi 710032, China; State Key Laboratory of Holistic Integrative Management of Gastrointestinal Cancers, Department of Pathology, Xijing Hospital and School of Basic Medicine, Fourth Military Medical University, Xi’an, Shaanxi 710032, China; State Key Laboratory of Holistic Integrative Management of Gastrointestinal Cancers, Department of Pathology, Xijing Hospital and School of Basic Medicine, Fourth Military Medical University, Xi’an, Shaanxi 710032, China; State Key Laboratory of Holistic Integrative Management of Gastrointestinal Cancers, Department of Pathology, Xijing Hospital and School of Basic Medicine, Fourth Military Medical University, Xi’an, Shaanxi 710032, China; State Key Laboratory of Holistic Integrative Management of Gastrointestinal Cancers, Department of Pathology, Xijing Hospital and School of Basic Medicine, Fourth Military Medical University, Xi’an, Shaanxi 710032, China; Department of Clinical Diagnosis, Tangdu Hospital, Fourth Military Medical University, Xi’an, Shaanxi 710038, China; State Key Laboratory of Holistic Integrative Management of Gastrointestinal Cancers, Department of Pathology, Xijing Hospital and School of Basic Medicine, Fourth Military Medical University, Xi’an, Shaanxi 710032, China; State Key Laboratory of Holistic Integrative Management of Gastrointestinal Cancers, Department of Pathology, Xijing Hospital and School of Basic Medicine, Fourth Military Medical University, Xi’an, Shaanxi 710032, China; State Key Laboratory of Holistic Integrative Management of Gastrointestinal Cancers, Department of Pathology, Xijing Hospital and School of Basic Medicine, Fourth Military Medical University, Xi’an, Shaanxi 710032, China; Department of Neurosurgery, the Second Affiliated Hospital of Xi’an Jiaotong University, Xi’an, Shaanxi 710004, China; Department of Pathology, School of Basic Medical Sciences, Ningxia Medical University, Yinchuan, Ningxia 750004, China; Institute of Analytical Chemistry and Instrument for Life Science, The Key Laboratory of Biomedical Information Engineering of Ministry of Education, School of Life Science and Technology, Xi’an Jiaotong University, Xi’an, Shaanxi 710049, China; State Key Laboratory of Holistic Integrative Management of Gastrointestinal Cancers, Department of Pathology, Xijing Hospital and School of Basic Medicine, Fourth Military Medical University, Xi’an, Shaanxi 710032, China; State Key Laboratory of Holistic Integrative Management of Gastrointestinal Cancers, Department of Pathology, Xijing Hospital and School of Basic Medicine, Fourth Military Medical University, Xi’an, Shaanxi 710032, China; State Key Laboratory of Holistic Integrative Management of Gastrointestinal Cancers, Department of Pathology, Xijing Hospital and School of Basic Medicine, Fourth Military Medical University, Xi’an, Shaanxi 710032, China

**Keywords:** α-ketoglutarate, IDH1 mutation, OGDH, l, -glutamine, gliomas

## Abstract

Isocitrate dehydrogenase (IDH) mutations frequently occur in lower-grade gliomas and secondary glioblastomas. Mutant IDHs exhibit a gain-of-function activity, leading to the production of D-2-hydroxyglutarate (D-2HG) by reducing α-ketoglutarate (α-KG), a central player in metabolism and epigenetic modifications. However, the role of α-KG homeostasis in IDH-mutated gliomagenesis remains elusive. In this study, we found that low expression of oxoglutarate dehydrogenase (OGDH) was a common feature in IDH-mutated gliomas, as well as in astrocytes. This low expression of OGDH resulted in the accumulation of α-KG and promoted astrocyte maturation. However, *IDH1* mutation significantly reduced α-KG levels and increased glutaminolysis and DNA/histone methylation in astrocytes. These metabolic and epigenetic alterations inhibited astrocyte maturation and led to cortical dysplasia in mice. Moreover, our results also indicated that reduced OGDH expression can promote the differentiation of glioma cells, while *IDH1* mutations impeded the differentiation of glioma cells with low OGDH by reducing the accumulation of α-KG and increasing glutaminolysis. Finally, we found that l-glutamine increased α-KG levels and augmented the differentiation-promoting effects of AGI5198, an *IDH1*-mutant inhibitor, in *IDH1*-mutant glioma cells. Collectively, this study reveals that low OGDH expression is a crucial metabolic characteristic of IDH-mutant gliomas, providing a potential strategy for the treatment of IDH-mutant gliomas by targeting α-KG homeostasis.

## Introduction

Isocitrate dehydrogenase (IDH) mutations predominantly occur in brain tumors, and the frequency of IDH mutation in lower-grade gliomas (LGGs) is more than 90% [1]. Mutant IDHs have gain-of-function activity in the conversion of α-ketoglutarate (α-KG) to D-2-hydroxyglutarate (D-2HG). It is widely established that D-2HG functions as an oncometabolite and affects epigenetic modifications by competitively inhibiting α-KG-dependent dioxygenases [2–5]. Owing to D-2HG production, IDH mutations naturally affect the homeostasis of α-KG, a crucial intermetabolite of the tricarboxylic acid (TCA) cycle. However, the role of α-KG homeostasis in IDH-mutated gliomagenesis remains unclear.

In the TCA cycle, α-KG is generated from isocitrate via oxidative decarboxylation, catalyzed by IDH, and then decarboxylated to form succinyl-CoA by oxoglutarate dehydrogenase (OGDH), a rate-limiting enzyme in the TCA cycle. This central metabolic junction allows for the anaplerotic production of α-KG from glutamate (Glu), either via oxidative deamination mediated by Glu dehydrogenase (GLUD) [6] or transamination reactions coupled with the production of other amino acids [7, 8]. In addition, disturbing α-KG homeostasis can affect the activities of α-KG-dependent dioxygenases, such as Jumonji C-domain lysine demethylases (JmjC-KDMs) and ten-eleven translocation (TET) DNA cytosine-oxidizing enzymes [9–11]. Alterations in α-KG-related metabolism potentially influence the acquisition of a cell-specific identity and the response to environmental signals by modulating the activity of α-KG-dependent dioxygenases [11]. Therefore, α-KG acts as a critical intermetabolite that connects metabolism and epigenetics, ultimately influencing cell fate.

In the brain, the expression patterns of several critical metabolic enzymes are reciprocally complementary between astrocytes and neurons, fulfilling the requirements of their different physiologic functions via several metabolite shuttles [12–14], such as the glutamine (Gln)−Glu cycle. In astrocytes, the entry of pyruvate into the TCA cycle is limited due to pyruvate dehydrogenase inactivation [15–17], whereas the expression of GLUD is significantly high, which guarantees sufficient α-KG generation to replenish the TCA cycle via Glu deamination [18–20]. In IDH-mutant gliomas, glutaminolysis is commonly augmented owing to massive D-2HG production and α-KG consumption [21, 22]. On the contrary, the transamination process via branched chain amino acid (BCAA) transaminases (BCATs), which consumes α-KG to produce glutamate, is diminished by downregulating the expression and activity of BCATs [7, 23]. Therefore, IDH-mutant glioma and its originating cells (astrocytes) exhibit unique feature in α-KG metabolism. However, whether the disturbed α-KG homeostasis is involved in the development of IDH-mutant gliomas needs further investigation.

To understand how IDH mutation facilitates the malignant transformation of normal astrocytes, we focused on studying the regulation of α-KG homeostasis by IDH mutation in both astrocytes and glioma cells. Our findings reveal that low expression of OGDH is a common metabolic characteristic of IDH-mutated gliomas and mature astrocytes. In both astrocytes and glioma cells with low OGDH expression, the IDH mutation markedly disrupts α-KG homeostasis, resulting in metabolic reprogramming, epigenetic alterations, and differentiation blocks. These alterations lead to cortical dysplasia in mice but are not evident in glioma cells with higher OGDH expression. Importantly, our results suggest that targeting α-KG homeostasis could be a potential approach for treating IDH-mutant gliomas with low expression of OGDH.

## Results

### Oxoglutarate dehydrogenase expression is low in isocitrate dehydrogenase-mutated gliomas

**Figure 1:**
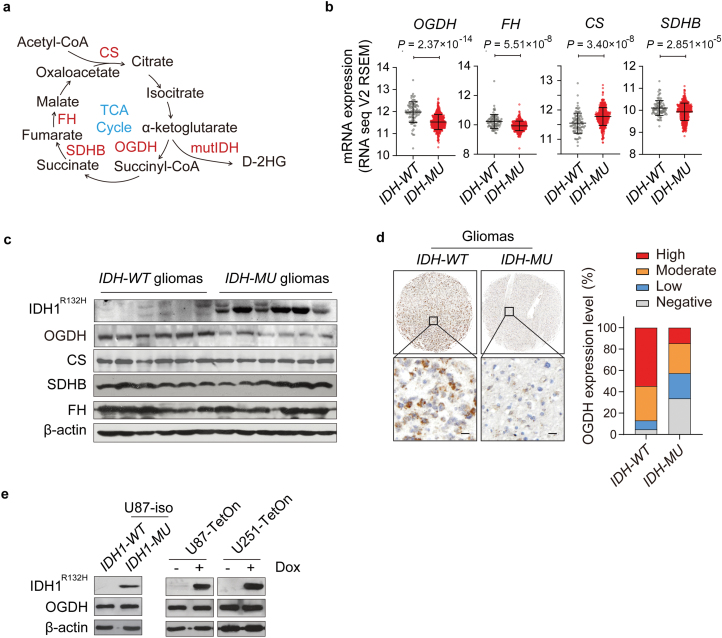
OGDH expression is low in IDH-mutated gliomas. (a) Schematic representation of oxidative decarboxylation of α-KG by OGDH. mutIDH, mutant IDH; D-2HG, D-2-hydroxyglutarate; TCA cycle, tricarboxylic acid cycle. (b) mRNA levels of *OGDH*, *FH*, *CS*, and *SDHB* in *ID*H-WT** and *ID*H-MU** LGGs were analyzed according to TCGA data (*n* = 92 in *ID*H-WT** group, *n* = 416 in *ID*H-MU** group). (c) OGDH, CS, SDHB, and FH protein levels in *ID*H-WT** and *ID*H-MU** glioma tissues, *n* = 6 for each group. (d) IHC staining (left) and statistical analysis (right) of OGDH in *ID*H-WT** and *ID*H-MU** glioma tissues. Scale bars are 20 μm, *n* = 84 in *I*DH-WT** group, and *n* = 89 in *ID*H-MU** group. (e) Western blot analysis showing OGDH protein expression in *ID*H1-WT** and *ID*H1-MU** U87 isogenic cells (U87-iso) (left), and U87-Teton and U251-Teton glioma cells with Dox-inducible overexpression of mutant *IDH1* (right). Biologically independent experiments were repeated three times.

OGDH functions as one of the major rate-limiting enzymes of the TCA cycle [24], catalyzing the decarboxylation of α-KG to generate succinyl-CoA (Fig. 1a). By analyzing The Cancer Genome Atlas (TCGA) data, we found that *OGDH* mRNA levels were significantly downregulated in IDH-mutated gliomas compared with other TCA cycle enzymes, including citrate synthase (CS), succinate dehydrogenase complex subunit B (SDHB), and fumarate hydratase (FH) (Fig. 1b; Supplementary Fig. S1a). Immunoblotting and immunohistochemical (IHC) staining confirmed that OGDH expression was remarkably low in IDH-mutated gliomas (Fig. 1c and d). It is well-established that patients with IDH-mutated gliomas have a significantly better prognosis. Our findings from the TCGA database also indicated that patients with low OGDH expression tended to have better prognostic characteristics (Supplementary Fig. S1b), suggesting that the favorable prognosis in glioma patients with low OGDH expression may be attributed to the accompanying IDH mutation. Low OGDH expression should be regarded as a metabolic hallmark of IDH-mutated gliomas. However, our results showed that OGDH expression remained unchanged both in *IDH1* isogenic mutant U87 cells (Fig. 1e left; Supplementary Fig. S1c) and in U87 and U251 cells with the doxycycline (Dox)-inducible overexpression of mutant *IDH1* (Fig. 1e right; Supplementary Fig. S1c). These results collectively indicate that OGDH expression is consistently low in IDH-mutated gliomas.

### Low oxoglutarate dehydrogenase expression promotes astrocyte maturation and glioma cell differentiation by elevating α-KG levels

**Figure 2:**
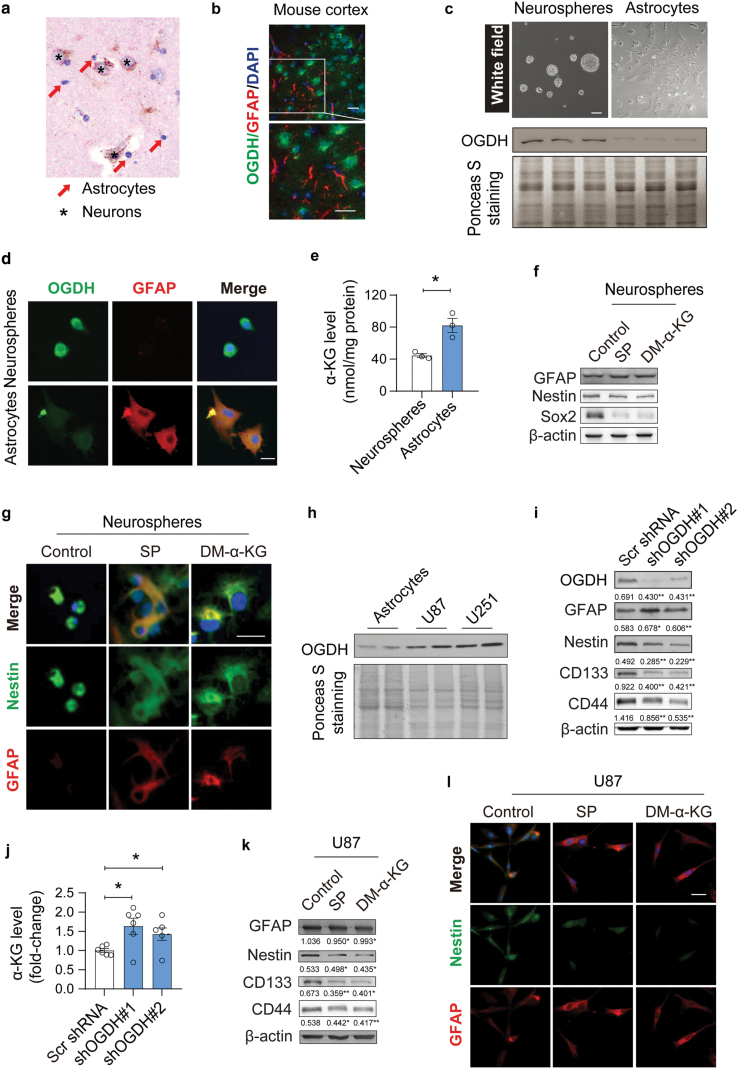
Low OGDH expression promotes astrocyte maturation and glioma cell differentiation by elevating α-KG levels. (a) IHC staining of OGDH in brain tissues. Red arrowheads, astrocytes; black asterisk, neurons. Scale bars are 20 μm. (b) IF staining of OGDH and GFAP in the cerebral cortex. Scale bars are 50 μm (top) and 20 μm (bottom), respectively. (c) Representative images of neurospheres (top left) and astrocytes differentiated from neurospheres (top right). Scale bars are 50 μm. The expression of OGDH is lowered in astrocytes (mild and bottom), *n* = 3. (d) Expression of OGDH and GFAP in the neurosphere cells and astrocytes in c, detected via IF staining. Two independent experiments were performed. Scale bars are 20 μm. (e) α-KG levels in the neurospheres and astrocytes, *n* = 3. (f) Expression of GFAP, Nestin, and Sox2 in neurosphere cells after treatment with SP (an OGDH inhibitor) and DM-α-KG. (g) IF staining of GFAP and Nestin in the neurosphere cells treated with SP, DM-α-KG, and DMSO (control). Scale bars are 12.5 μm. (h) Expression of OGDH in astrocytes and U87 and U251 cells, *n* = 2. (i) Expression of OGDH, GFAP, Nestin, CD133, and CD44 in *ID*H1-WT** U87 cells with silenced OGDH expression. (j) Quantitation of α-KG levels in *ID*H1-WT** U87 cells with silenced OGDH, *n* = 6. (k) Expression of GFAP, Nestin, CD133, and CD44 in *ID*H1-WT** U87 cells treated with SP, DM-α-KG, and DMSO (control). (l) IF staining of GFAP and Nestin in *ID*H1-WT** U87 cells treated with SP, DM-α-KG, and DMSO (control). Biologically independent experiments were repeated three times in b, f, g, i, k, and l. Data are represented as mean ± SEM. Statistical significance was determined using unpaired Student’s *t*-test (e) and one-way ANOVA (j), respectively. ^*^P < 0.05, ^**^P < 0.01, ^***^P < 0.001; ns, not significant.

Due to the fact that glioma originates from glial cells in the central nervous system (CNS), we examined the expression of OGDH in the brain. IHC staining results showed that OGDH was almost undetectable in astrocytes but was abundant in neurons (Fig. 2a). Immunofluorescence (IF) staining also demonstrated that OGDH was not expressed in glial fibrillary acidic protein (GFAP, a marker of mature astrocytes)-positive mouse astrocytes (Fig. 2b). We then cultured mouse neurospheres (which mainly contain neural stem cells (NSCs)) and differentiated them into astrocytes as described in “Materials and methods” section, and the immunoblotting results showed that OGDH expression was significantly lower in astrocytes compared with that in neurospheres (Fig. 2c). IF staining results also showed that OGDH was highly expressed in neurospheres but decreased upon differentiation into astrocytes (Fig. 2d; Supplementary Fig. S2a). These data suggest that low OGDH is an intrinsic feature of mature astrocytes. Interestingly, we also found that OGDH levels were relatively low in the bone marrow, spleen, and brain, from which IDH-mutant tumors commonly originate (Supplementary Fig. S2b).

Next, we sought to investigate the consequence of low OGDH expression in astrocytes and IDH-mutant gliomas. OGDH functions as a rate-limiting enzyme in α-KG catabolism in the TCA cycle, and low OGDH expression might alter α-KG homeostasis. Here, we found that α-KG levels were higher in mature astrocytes with low OGDH expression compared with that in neurospheres (Fig. 2e). To determine whether α-KG accumulation affects astrocyte maturation, the neurospheres were treated with dimethyl-α-KG (DM-α-KG, a cell-permeable α-KG) and succinyl phosphonate (SP, an OGDH inhibitor). The immunoblotting results indicated that both DM-α-KG and SP increased the expression of GFAP but inhibited the expression of Nestin and SRY (sex determining region Y)-box transcription factor 2 (Sox2) (markers of nervous system stem cells) (Fig. 2f). Moreover, IF staining of GFAP and Nestin also showed that both compounds promoted astrocyte maturation (Fig. 2g), indicating that low OGDH expression promotes astrocyte maturation by increasing α-KG contents.

In glioma cells (U87 and U251), we found that the OGDH expression was relatively higher than in astrocytes (Fig. 2h), which was consistent with observations based on human *ID*H*-WT* glioma samples. Then, we knocked down OGDH in U87 and U251 glioma cells using lentivirus-mediated short-hairpin RNA (shRNA). Consequently, we found an increase in the expression of GFAP, but a decrease in the expression of Nestin and two markers of glioma stem cells, CD133 and CD44 (Fig. 2i; Supplementary Fig. S2c), indicating that low expression of OGDH promotes glioma cell differentiation. Moreover, our results proved that α-KG levels were correspondingly increased in OGDH-silenced U87 cells (Fig. 2j). In addition, both DM-α-KG and SP promoted the differentiation of glioma cells through elevated GFAP expression and reduced Nestin, CD133, and CD44 expression (Fig. 2k and l), suggesting that α-KG accumulation can promote the differentiation of glioma cells.

### Astrocyte-specific knockin of mutant *IDH1* impedes astrocyte maturation and cortex development in mice

**Figure 3:**
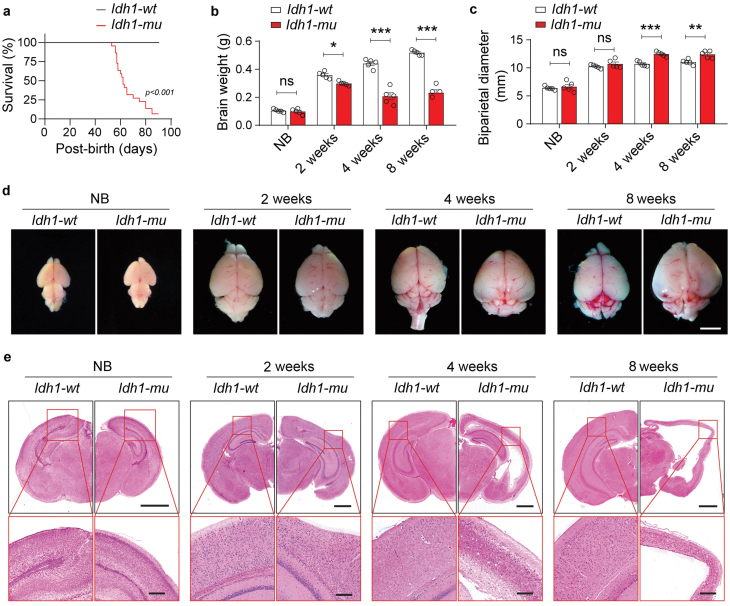
Astrocyte-specific mutant *IDH1* knock-in impedes cortex development in mice. (a) Survival curves of *Id*h1-mu** (*n* = 20) and *Id*h1-wt** (*n* = 20) mice. Statistical significance was determined using a log-rank (Mantel–Cox) test. (b and c) The brain weights (b) and biparietal diameters (c) of *Id*h1-wt** and *Id*h1-mu** mice in NBs and at 2, 4, and 8 weeks of age. (d and e) Macroscopic views (d) and H&E staining (e) of brain sections in *Id*h1-wt** and *Id*h1-mu** mice at the indicated ages. Scale bars are 5 mm (d) and 1 mm (e), respectively. Data are represented as mean ± SEM. Statistical significance was determined using one-way ANOVA (b and c). ^*^P < 0.05, ^**^P < 0.01, ^***^P < 0.001; ns, not significant.

To investigate the functional role of IDH mutations during astrocyte differentiation *in vivo*, we generated astrocyte-specific *mutant-Id*h*1* knock-in (*Id*h1*-mu*) mice. The results showed that the survival time of *Id*h1*-mu* mice was only 2 months (Fig. 3a). We found that compared with wild-type (*Id*h1*-wt*) mice, the brain weights of *Id*h*1-mu* mice decreased at 2 weeks of age and significantly exacerbated at 4 and 8 weeks of age (Fig. 3b), but the biparietal diameters were only slightly increased (Fig. 3c). Further anatomical and histological analyses showed that the brains of newborn (NB) *Id*h1*-mu* and *Id*h1*-wt* mice were similar (Fig. 3d and e), but the cerebral cortex of *Id*h1*-mu* mice became thinner at 2 weeks of age, which became more severe at 4 and 8 weeks of age (Fig. 3d and e). No gliomas were found in the brains of *Id*h1*-mu* mice during the observation.

**Figure 4:**
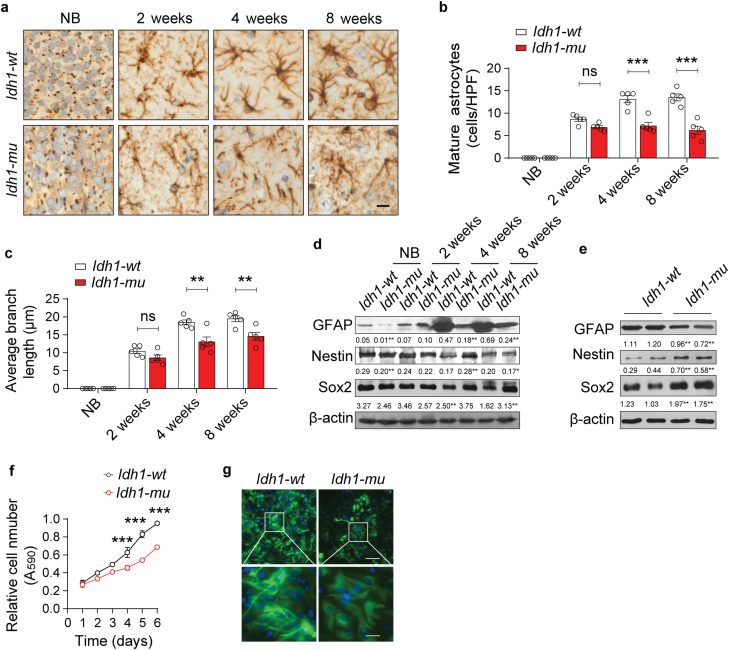
The *IDH1* mutation impedes the maturation of astrocytes. (a) The astrocytes in the cerebral cortex of *Id*h1-wt** and *Id*h1-mu** mice were detected by performing GFAP staining. Scale bars are 10 μm. Five independent experiments were performed. (b) The densities of mature astrocytes (with more than three branches) were calculated based on high-power fields (HPFs) at different ages, *n* = 5. (c) Average branch lengths of *Id*h1-wt** and *Id*h1-mu** astrocytes in the cortex section, *n* = 5. (d) Immunoblotting for GFAP, Nestin, and Sox2 in the brains of *Id*h1-wt** and *Id*h1-mu** mice at the indicated ages. Five independent experiments were performed. (e) Expression of GFAP, Nestin, and Sox2 in *Id*h1-wt** and *Id*h1-mu** astrocytes. (f) Growth curves of *Id*h1-wt** and *Id*h1-mu** astrocytes. (g) IF staining of GFAP in *Id*h1-wt** and *Id*h1-mu** astrocytes. Scale bars are 200 μm (top) and 50 μm (bottom), respectively. Data are represented as mean ± SEM. Statistical significance was determined using one-way ANOVA (b, c, f). ^*^P < 0.05, ^**^P < 0.01, ^***^P < 0.001; ns, not significant.

Next, we investigated the astrocyte maturation in *Id*h*1-mu* mice. GFAP immunostaining results showed that the *IDH1* mutation hindered astrocyte maturation in the cortex of *Id*h*1-mu* mice (Fig. 4a). This was evident based on the decreased densities of mature astrocytes (Fig. 4b) and the shortened length of astrocyte foot processes (Fig. 4c). Immunoblotting results also showed that GFAP expression was decreased but Nestin and Sox2 levels were elevated in the cortex of *Id*h*1-mu* mice at various ages, compared with wild-type group (Fig. 4d). Furthermore, we isolated and cultured mouse astrocytes. Results in astrocytes from *Id*h*1-mu* mice showed a decrease in GFAP expression and an increase in Nestin and Sox2 expression (Fig. 4e). In addition, the growth of *Id*h*1-mu* astrocytes was slower than that of *Id*h*1-wt* astrocytes (Fig. 4f). IF staining further showed decreased expression of GFAP and impeded astrocyte maturation in *Id*h*1-mu* astrocytes (Fig. 4g). Collectively, these results indicate that the *IDH1* mutation impedes astrocyte maturation and causes cortex mal-development in mice.

### The *IDH1* mutation significantly disturbs α-KG homeostasis in astrocytes with low oxoglutarate dehydrogenase expression

**Figure 5:**
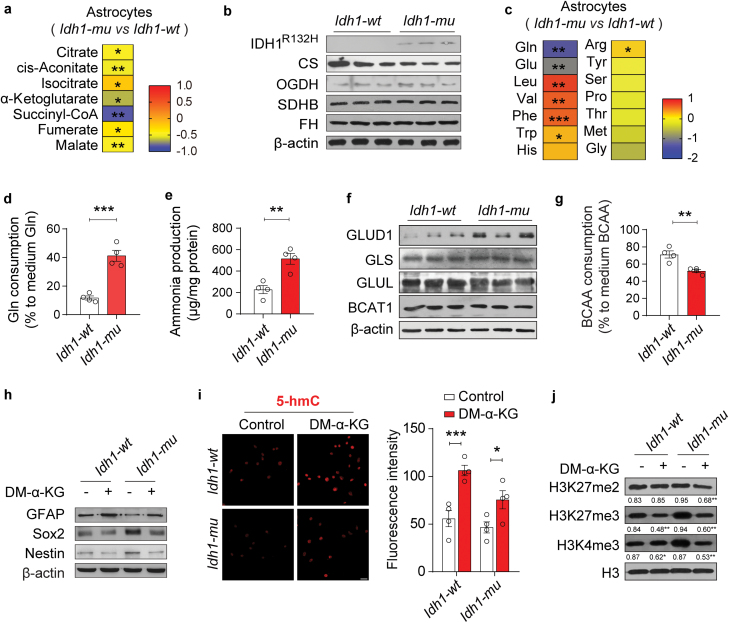
The *IDH1* mutation disrupts α-KG homeostasis in astrocytes. (a) Changes in TCA cycle intermetabolites in *Id*h1-mu** astrocytes (normalized to the levels in the *Id*h1-wt** astrocytes), *n* = 6. (b) Expression of TCA cycle enzymes, including CS, OGDH, SDHB, and FH, in *Id*h1-mu** and Id*h1-wt* astrocytes, *n* = 3. (c) Levels of representative amino acids in *Id*h1-mu** astrocytes (normalized to the levels in *Id*h1-wt** astrocytes), *n* = 6. (d and e) Gln consumption (d) amd ammonia production (e) in *Id*h1-mu** and *Id*h1-wt** astrocytes, *n* = 4. (f) Expression of GLUD1, GLS, GLUL, and BCAT1 in *Id*h1-mu** and *Id*h1-wt** astrocytes, *n* = 3. (g) BCAA consumption in *Id*h1-mu** and *Id*h1-wt** astrocytes, *n* = 4. (h–j) After the *Id*h1-mu** and *Id*h1-wt** astrocytes were treated with DM-α-KG or DMSO, the levels of GFAP, Nestin, and Sox2 (h), and H3K27me2, H3K27me3, and H3K4me3 (j) were detected through immunoblotting, and changes in 5-hmC were detected by IF staining (i). Scale bars are 20 μm. Biologically independent experiments were repeated three times in h–j. Data are represented as the mean ± SEM. Statistical significance was determined using an unpaired Student’s *t*-test (d and e), Mann–Whitney test (g), and one-way ANOVA (i). ^*^P < 0.05, ^**^P < 0.01, ^***^P < 0.001; ns, not significant.

As an intermetabolite, α-KG is involved in several crucial metabolic pathways. Therefore, we further examined the α-KG-related metabolic pathways in *Id*h1*-mu* astrocytes. Metabolomic profiling of TCA cycle intermetabolites showed that α-KG levels were decreased by approximately 50% in *Id*h1*-mu* astrocytes, and succinyl-CoA levels were also significantly decreased, compared with that in wild-type group (Fig. 5a). However, the protein levels of representative TCA cycle enzymes, such as OGDH, SDHB, CS, and FH, were not significantly altered in the *Id*h*1-mu* astrocytes (Fig. 5b). In astrocytes, α-KG is closely related to the transamination and deamination of amino acids. Here, we found that the Gln and Glu levels were significantly lower in *Id*h*1-mu* astrocytes compared with wild-type group, but BCAAs, such as leucine and valine, were elevated (Fig. 5c), implying that the *IDH1* mutation could increase glutaminolysis but inhibited BCAA transamination to compensate for α-KG consumption. Our results further proved that the *IDH1* mutation could augment Gln consumption and elevate ammonia production in astrocytes (Fig. 5d and e). Immunoblotting results also showed that the *IDH1* mutation increased the expression of GLUD1 in astrocytes, without altering the expression of glutaminase (GLS) and Gln synthase (GLUL) (Fig. 5f). Moreover, BCAA consumption was reduced in *Id*h*1-mu* astrocytes (Fig. 5g), although BCAT1 expression was not altered by the *IDH1* mutation (Fig. 5f).

In addition to metabolic pathways, intracellular α-KG homeostasis also affects the activities of α-KG-dependent dioxygenases, such as JmjC-KDMs and TETs; thus, we treated *Id*h*1-mu* astrocytes with DM-α-KG. Intriguingly, we found that DM-α-KG increased GFAP expression, and inhibited the expression of Nestin and Sox2 more significantly in *Id*h*1-mu* astrocytes when compared with wild-type group (Fig. 5h). Our results also showed that the IDH mutation decreased 5-hmC (5-hydroxymethylcytosine) levels in *Id*h*1-mu* astrocytes, which could be restored by DM-α-KG (Fig. 5i). Moreover, compared with wild-type group, H3K27me3 and H3K4me3 levels were elevated in *Id*h*1-mu* astrocytes, which could be reversed by DM-α-KG (Fig. 5j). Collectively, these results indicate that the *IDH1* mutation significantly disturbs α-KG homeostasis in astrocytes with low OGDH expression and consequently alters α-KG-related amino acid metabolism and epigenetic modifications.

### The *IDH1* mutation significantly affects α-KG-related epigenetic alterations and metabolic reprogramming in glioma cells with low oxoglutarate dehydrogenase expression

**Figure 6:**
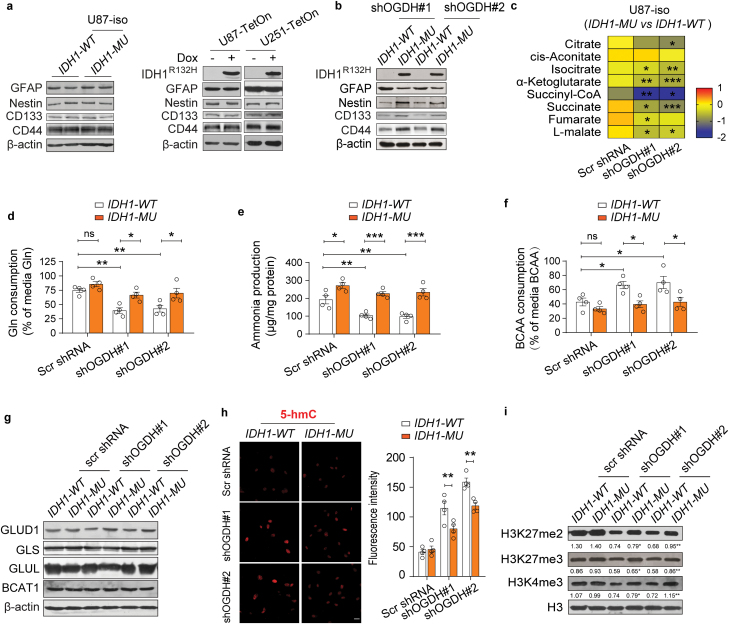
The *IDH1* mutation blocks the differentiation of glioma cells with low OGDH expression by reducing α-KG levels. (a) Western blot showing GFAP, Nestin, CD133, and CD44 protein expression in *ID*H1-MU** U87 isogenic cells (left), U87-Teton and U251-Teton glioma cells with Dox-inducible overexpression of mutant *IDH1* (right). (b) Protein levels of GFAP, Nestin, CD133, and CD44 in *ID*H1-WT** and *ID*H1-MU** U87 cells after OGDH expression was knocked down. (c) Changes in TCA cycle intermetabolites in *ID*H1-MU** U87 cells with silenced OGDH (normalized to the levels in the wild-type groups), *n* = 6. (d–f) Gln consumption (d), ammonia production (e), and BCAA consumption (f) in *ID*H1-WT** and *ID*H1-MU** U87 cells with silenced OGDH expression, *n* = 4. (g) Protein levels of GLUD1, GLS, GLUL, and BCAT1 in *ID*H1-WT** and *ID*H1-MU** U87 cells with silenced OGDH expression. (h and i) Representative images of IF staining for 5-hmC (h) and immunoblotting for H3K27me2, H3K27me3, and H3K4me3 (i) in *ID*H1-WT** and *ID*H1-MU** U87 cells with silenced OGDH expression. Scale bars, 20 μm. Biologically independent experiments were repeated three times in a, b, g, and i. Data are represented as the mean ± SEM. Statistical significance was determined using one-way ANOVA (c–f, h). ^*^P < 0.05, ^**^P < 0.01, ^***^P < 0.001; ns, not significant.

The *IDH1* mutation impeded the maturation of astrocytes with low OGDH, but we found that it did not alter the expression of GFAP, Nestin, CD133, and CD44 in U87 and U251 glioma cells (Fig. 6a). Interestingly, we found that the *IDH1* mutation could reduce GFAP expression and increase the expression of Nestin, CD133, and CD44 in OGDH-silenced U87 cells (Fig. 6b). The IDH mutation was believed to epigenetically manipulate gene transcription through the production of high levels of D-2HG; yet, OGDH knockdown did not increase D-2HG levels in IDH-mutated cells (Supplementary Fig. S3a). Through metabolomics, we found that the reduction of TCA metabolites in IDH-mutated glioma cells with low OGDH expression was more significant compared to that in control cells (Fig. 6c), but TCA cycle enzymes were not significantly altered (Supplementary Fig. S3b). These results indicate that the *IDH1* mutation significantly disturbs α-KG homeostasis in glioma cells with low OGDH expression and consequently blocks their differentiation.

In U87 cells, silencing OGDH expression reduced glutaminolysis and the ammonia product but increased BCAA catabolism, which could alleviate α-KG accumulation (Fig. 6d–f). Interestingly, compared with wild-type group, the *IDH1* mutation resulted in a more significant increase in Gln consumption and ammonia production in U87 cells when OGDH was knocked down (Fig. 6d and e). In addition, the *IDH1* mutation resulted in a more significant decrease in BCAA consumption in OGDH-silenced U87 cells (Fig. 6f). These metabolic alterations implied that the effects of the *IDH1* mutation on disturbing α-KG-related amino acid metabolism were more significant in glioma cells with low OGDH expression. Moreover, immunoblotting results also indicated that the *IDH1* mutation increased GLUD1 expression in OGDH-silenced U87 cells (Fig. 6g), which was consistent with the changes observed in IDH-mutated gliomas (Supplementary Fig. S3c and d). Consistently, IF staining results showed that the *IDH1* mutation significantly decreased 5-hmC levels in OGDH-silenced glioma cells (Fig. 6h), and immunoblotting data showed that the *IDH1* mutation significantly elevated H3K27me3 and H3K4me3 levels in the OGDH-silenced U87 cells (Fig. 6i). These results indicate that the *IDH1* mutation can remarkably disturb α-KG homeostasis in glioma cells with low OGDH expression and ultimately block their differentiation.

### Glutamine increases α-KG levels in glioma cells with low oxoglutarate dehydrogenase expression and augments the effects of AGI5198

**Figure 7:**
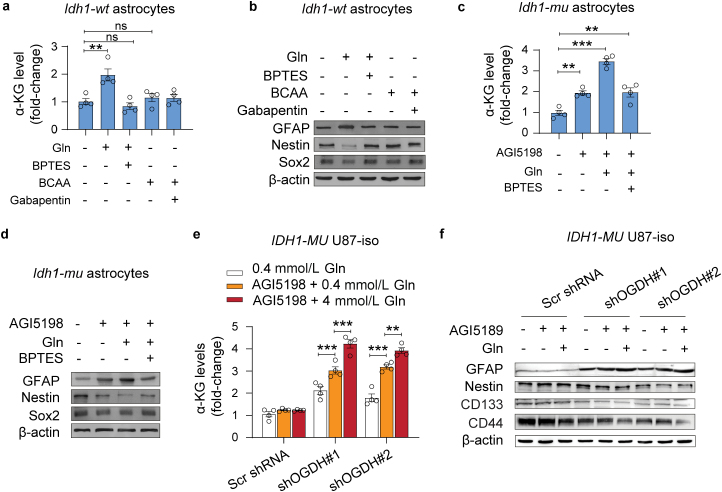
Gln elevates α-KG levels in glioma cells with low OGDH expression and augments the effects of AGI5198. (a and b) α-KG levels (a) and GFAP, Nestin, and Sox2 expression (b) in *Id*h1-wt** astrocytes treated with BPTES (a GLS inhibitor) and gabapentin (a BCAT inhibitor) in the medium with l-Gln or BCAAs, respectively. (c and d) α-KG levels (c) and the expression of GFAP, Nestin, and Sox2 (d) in *Id*h1-mu** astrocytes treated with AGI5198 (an *ID*H1**^*R132H*^ mutation inhibitor) and BPTES in the medium with l-Gln. (e and f) α-KG levels (e) and the expression of GFAP, Nestin, CD133, and CD44 (f) in *ID*H1-MU** U87 isogenic cells with silenced OGDH expression after treatment with AGI5198 in the presence of low (0.4 mmol/L) or high (4 mmol/L) concentrations of Gln, respectively; *n* = 4 in a, c, and e. Biologically independent experiments were repeated three times in b, d, and f. Data are represented as the mean ± SEM. Statistical significance was determined using one-way ANOVA (a, c, e). ^*^P < 0.05, ^**^P < 0.01, ^***^P < 0.001; ns, not significant.

Glutaminolysis and BCAA catabolism are closely associated with intracellular α-KG turnover; accordingly, we treated the primary astrocytes with Gln and BCAAs. The results showed that Gln could increase α-KG levels in astrocytes, which could be inhibited by bis-2-(5-phenylacetamido-1,2,4-thiadiazol-2-yl)ethyl sulfide (BPTES, a GLS inhibitor) (Fig. 7a). However, BCAAs and gabapentin (a BCAT inhibitor) did not alter the α-KG levels in astrocytes (Fig. 7a). Consistently, immunoblotting results indicated that Gln could promote astrocyte maturation by increasing GFAP expression and decreasing Nestin and Sox2 expression, but BCAAs and gabapentin did not have this effect (Fig. 7b). These results indicate that glutaminolysis could affect α-KG homeostasis in astrocytes.

Mutant *IDH1/2* inhibitors have been used to treat IDH-mutant cancers [25]. Here, we found AGI5198, an IDH1^R132H^ inhibitor, increased α-KG levels in *Id*h1*-mu* astrocytes. Moreover, Gln intensified the increase in intracellular α-KG levels in AGI5198-treated *Id*h1*-mu* astrocytes, which was blocked by BPTES (an inhibitor of GLS) (Fig. 7c). More interestingly, Gln and AGI5198 also synergistically increased GFAP expression, but decreased Nestin and Sox2 expression, in *Id*h1*-mu* astrocytes (Fig. 7d). In *ID*H1*-mutant* U87 cells with silenced OGDH expression, AGI5198 and Gln synergistically increased α-KG levels (Fig. 7e) and increased GFAP expression, but decreased Nestin, CD133, and CD44 expression (Fig. 7f). These results indicate that Gln could increase α-KG levels in glioma cells with low OGDH expression and augment the effects of the mutant IDH inhibitor.

## Discussion

IDH mutations are commonly found in tumors from various tissues, but they are predominantly observed in brain tumors [26, 27]. The introduction of the IDH mutation status into the WHO Classification of Tumors of the Central Nervous System guidelines has facilitated the diagnosis and classification of gliomas [28]. Unlike the effect of IDH mutations on the outcomes of other cancers, patients with IDH-mutated gliomas generally have a more favorable prognosis than those with IDH-wild-type gliomas [1], but treating IDH-mutated gliomas with mutant IDH inhibitors is controversial [29]. Therefore, genetic and metabolic backgrounds must be considered when studying the development and treatment of IDH-mutated gliomas.

Gliomas originate from glial cells in the CNS, including astrocytes and oligodendrocytes, which are dominant cell types in the brain and have crucial roles in supporting the nutrient acquisition and functions of neurons. In mature astrocytes, OGDH expression is low, which is crucial for their metabolic functions. This reduced OGDH expression results in the accumulation of α-KG and promotes Gln biosynthesis, critical for maintaining the Glu–Gln cycle between neurons and astrocytes [30]. Here, we observed a decrease in OGDH expression in astrocytes that differentiated from neurospheres. Low OGDH not only promotes the accumulation of α-KG in astrocytes but also drives the maturation of astrocytes. Previous research has shown that α-KG can facilitate the differentiation of pluripotent cells, such as human embryonic stem cells and mouse post-implantation epiblast embryonic stem cells [31, 32]. Thus, as an intrinsic feature of astrocytes, low OGDH expression drives astrocytes to mature through the regulation of α-KG turnover.

OGDH expression and activity are commonly upregulated to adapt to the increased glycolytic capacity of cancer cells [33–35]. Increased OGDH expression contributes to a heightened reliance on α-KG turnover, which can maintain respiration with normal electron transport chain and NAD^+^/NADH ratios or promote anabolic metabolism [36, 37]. Conversely, low OGDH expression leads to α-KG accumulation [24, 38–40], and knockdown of OGDH has been shown to stimulate tumor cell differentiation by increasing α-KG accumulation and inducing epigenetic modifications [40]. In our study, we observed that glioma cells exhibited higher OGDH levels than normal astrocytes and decreasing OGDH expression increased α-KG content and promoted glioma cell differentiation. Therefore, OGDH has a crucial role in regulating metabolism and the epigenetic signature of cancer cells, which mainly depends on affecting α-KG contents. GSCs are crucial in the formation, perpetuation, and recurrence of gliomas, and they share cellular and molecular characteristics with NSCs in neuronspheres [41, 42]. In our study, reducing OGDH promoted the differentiation of NSCs into astrocytes, as well as the differentiation of glioma cells. Therefore, we postulate that lowering OGDH expression is likely to diminish the stemness and encourage differentiation of GSCs, and targeting OGDH expression and activity could be a viable strategy in glioma treatment.

In IDH-mutated gliomas, we found that low OGDH expression is an intrinsic characteristic. Low OGDH expression promoted the differentiation of glioma cells by increasing α-KG levels. This effect could be reversed by IDH mutation independent of D-2HG production, although D-2HG is considered as an oncometabolite to regulate DNA/histone methylation, hypoxia signaling, and DNA repair, affecting the oncogenesis of IDH-mutated cancers [43]. Intracellular α-KG homeostasis is strictly governed by several metabolic enzymes, such as IDH, OGDH, transaminases, and GLUDs. We found that the IDH mutation exerted a more substantial impact on α-KG homeostasis in glioma cells with low OGDH expression, resulting in enhanced effects on α-KG-associated differentiation and epigenetic changes. These findings imply that low OGDH expression establishes a specific metabolic context in which the *IDH1* mutation can have a stronger impact on α-KG homeostasis. This might also contribute to the high incidence of IDH mutation in brains.

The disturbed α-KG homeostasis resulted in an increase in glutaminolysis and a decrease in transamination in glioma cells with low OGDH expression, which served as a compensatory mechanism to maintain α-KG turnover. Additionally, we observed that a mutant *IDH1* inhibitor significantly raised α-KG levels and aided the differentiation of *IDH1*-mutated glioma cells with low OGDH expression. Furthermore, Gln supplementation was found to further boost α-KG accumulation and enhance the effects of the mutant IDH inhibitor on differentiation. These findings highlight that Gln can bolster the effects of a mutant IDH inhibitor during the treatment of IDH-mutated gliomas.

In this study, no gliomagenesis was observed in the cortex of *IDH1*-mutant mice, which is consistent with previous reports [44, 45]. These results also suggest that D-2HG production and the disturbed α-KG homeostasis are not sufficient to initiate tumorigenesis, although they can reprogram metabolic pathways and alter epigenetic modifications. Recent studies have confirmed that *IDH1* mutations can trigger glioma formation in mice when combined with mutations in other key tumor suppressor genes, such as *TP53* (Tumor protein p53) and *ATRX* (alpha-thalassemia mental retardation X-linked) [46]. Exploring the potential impact of TP53 and ATRX changes on α-KG homeostasis in IDH-mutated gliomas to facilitate tumorigenesis will be an intriguing area of research.

**Figure 8:**
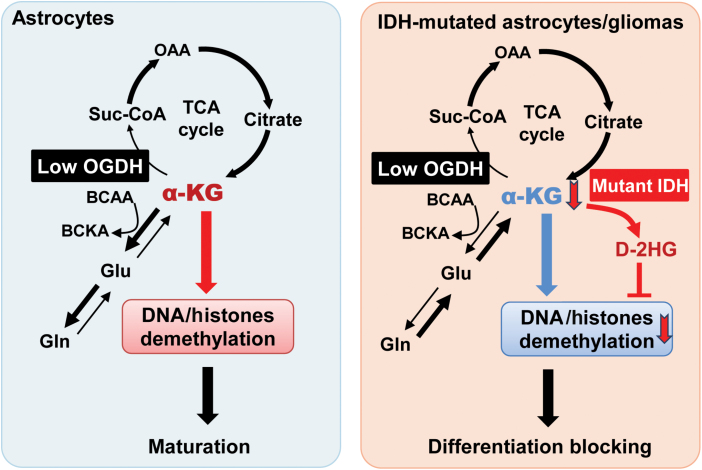
The *IDH1* mutation leads to metabolic and epigenetic alterations in astrocytes and gliomas with low OGDH expression by disturbing α-KG homeostasis. OGDH is expressed at lower levels in astrocytes and IDH-mutated gliomas. Low OGDH expression promotes the maturation of astrocytes by increasing α-KG levels. Elevated α-KG can increase Gln production and BCAA transamination and promote the demethylation of DNA/histones and astrocyte maturation. Interestingly, knockdown of OGDH facilitates the differentiation of glioma cells, and this effect can be reversed by an *IDH1* mutation. The *IDH1* mutation further reduces α-KG levels, particularly in glioma cells with low OGDH expression, which coincides with increased glutaminolysis, decreased BCAA transamination, and DNA/histone hypermethylation. These findings suggest that low OGDH expression establishes a specific α-KG metabolic context, wherein the *IDH1* mutation exerts a more significant impact on α-KG homeostasis, promoting glioma dedifferentiation.

In summary, we demonstrate that OGDH expression is diminished in mature astrocytes and IDH-mutant gliomas. Crucially, the *IDH1* mutation has a more pronounced effect on disturbing α-KG homeostasis in glioma cells with low OGDH expression. Moreover, the α-KG-related metabolic reprogramming and hypermethylation phenotype are intimately associated with the differentiation of *IDH1*-mutant gliomas (Fig. 8). Therefore, our results have significant implications for understanding metabolic remodeling in IDH-mutant gliomas and offer a potential metabolic target for treating this disease.

## Materials and methods

### Animals

The mutant *IDH1* conditional knock-in mice were bred with GFAP-Cre mice to produce *Id*h1**^**l*sl-R132H/+*^;*GFAP-Cre* (*Id*h*1-mu*) mice, and the *IDH1*^+/+^;GFAP-Cre (*Id*h1*-wt*) mice were utilized as control. The genotypes are detailed in Supplementary Fig. S4. The animals were allowed free access to water and a standard laboratory diet, a light–dark cycle was maintained for 12 h, and the temperature was 20–25°C. The animal studies were approved by the Animal Care Committees of The Fourth Military Medical University (China) (project number: IACUC-20180307).

### Glioma samples

Glioma tissues were collected from the Department of Neurosurgery of Tangdu Hospital, The Fourth Military Medical University. IDH mutations were determined by Sanger sequencing, and the results were approved by pathologists from the Department of Pathology at Xijing Hospital. The ethics committee of Tangdu Hospital of Fourth Military Medical University approved the procedure (project number: TDLL-202205-06).

### Cell lines and growth conditions

*IDH1-WT* (ATCC Lot: 63710285) and *IDH1*-mutant (**ID*H1**^**R132*H*^*) (ATCC Lot: 70004232) U87 isogenic cells were acquired from American Type Culture Collection (ATCC). U251 glioma cells procured from our laboratory were authenticated by short tandem repeat profiling, and mycoplasma contaminations were detected by PCR. Cell lines were incubated at 37°C in 5% CO_2_, using Dulbecco’s modified Eagle’s medium (DMEM) (Gibco, 12100046) supplemented with 10% fetal bovine serum (FBS) (Gibco, 10100147) as the standard culture medium. Conditional DMEM (Gibco, A1443001) containing 5.5 mmol/L glucose with or without 4 mmol/L Gln was used for the Gln, ammonia, and BCAA assays. BCAA (1 mmol/L l-isoleucine, 1 mmol/L l-leucine, 1 mmol/L l-valine, Sigma, I7403, L8912, V0513, respectively). 1 mmol/L SP (MCE, HY-12688A), 10 μmol/L BPTES (Topscience, T6791), 20 mmol/L gabapentin (Topscience, T0830), and 4 mmol/L DM-α-KG (Sigma, 349631) were added to the culture medium.

### Oxoglutarate dehydrogenase knockdown in glioma cells

The OGDH expression in U87 cells was silenced using shRNA. The shRNA fragments were subcloned into pLKO.1-TRC lentivirus vector (Addgene), and the target sequences of these shRNAs were: 5'-CAACAAGATGAAGAGCACCAA-3' (Scramble shRNA), 5'-GATCATGCAGTTCACAAATGA-3' (ShOGDH#1), 5'-GGAACAGATCTTCTGTCAATT-3' (ShOGDH#2), respectively. The lentiviral plasmids were co-transfected with psPAX2 and pVSV-G into HEK-293T cells. The recombinant virus-containing medium was collected and filtered after 72 h. Then, different glioma cells were infected with lentivirus in the presence of polybrene. After 72 h, the infected U87 cells were selected with puromycin, and OGDH expression in cells was determined by immunoblotting.

### Inducible expression of *IDH1*^*R132H*^ in glioma cells

For the inducible expression of mutant *IDH1* in glioma cells, the mutant *ID*H1**^**R*132H*^ was subcloned into pLVX-Tight-Neo vectors (Clontech) to generate the pLVX-Tight-*ID*H1**^*R132H*^-Neo lentiviral plasmid. pLVX-TetOn-Puro plasmid and pLVX-Tight-*ID*H1**^**R1*32H*^-Neo lentiviral plasmid were co-transfected with psPAX2 and pVSVG into HEK-293T cells to generate the lentivirus. The infected U87 and U251 cells were selected with puromycin and geneticin (G418) for 2 weeks, and the expression of **ID*H1**^*R132H*^* was confirmed by immunoblotting in the presence or absence of 10 µmol/L Dox.

### Astrocyte isolation and culture

Primary astrocytes were isolated from the cortex of NB mice (1-day pups). After stripping the meninges, the brains were mechanically dissociated into pieces and treated with 0.25% trypsin for 15 min at 37°C. After centrifugation at 1500 rpm, the isolated cells were collected and the floccules were further treated with 1 mg/L DNase I. The isolated pup glial cells were inoculated in 60-mm dishes and cultured in DMEM supplemented with 10% FBS. After confluence, the cultures were shaken for 24 h at 250 rpm to remove microglial cells and oligodendrocytes, and the floating cells were collected and used as astrocyte-enriched fractions. Cells were also cultured in a standard culture medium.

### Neurosphere culture and differentiation into astrocytes

The neurosphere cultures were prepared from cerebral hemispheres of postnatal day 0–2 mice. The meninges were removed, and the cerebral hemispheres dissected apart from the rest of the brain. The cerebral hemispheres were chopped into small pieces and trypsin dissociation solution was added, which were mixed well and incubated at 37°C for 1 h. Then, 1 mL trypsin inhibitor without FBS (TBD Cat #SC2013-G-E) was added, and the brain tissue was broken up into a milky white homogenate with no visible chunks. The cells were pelleted by centrifugation at 1000 rpm for 5 min. The supernatant was removed and the cells were resuspended in 1 mL complete NSC medium, to culture medium defined, serum-free NSC medium containing DMEM/F12 supplemented with human recombinant epidermal growth factor (20 ng/mL; Novoprotein, Cat #CO29), basic fibroblast growth factor (20 ng/mL; Novoprotein, Cat #CH88), and B27 (Invitrogen, 12587-010). The neurospheres were cultured on a bacteriological grade 100-mm Petri dish in a 5% CO_2_, 37°C incubator. Typically, the neurospheres were passaged every 5–7 days.

A pool of neurospheres was collected by centrifuging at 800 rpm for 3 min. The neurospheres were resuspended in 1 mL of astrocyte culture medium in a PDL-coated culture dish. Ten percent of FBS promoted NSC differentiation toward the astrocyte lineage. The neurospheres attached to the surface and began to differentiation into astrocyte lineage. When the astrocyte culture became 50–60% confluent, the astrocyte culture was passaged and plated on new petri dishes.

### Histological analysis

For the histological analysis, the brains from *Id*h1*-wt* and *Id*h1*-mu* mice at different ages were collected. The brains were fixed with 4% paraformaldehyde (PFA), embedded in paraffin, and stained with hematoxylin and eosin (H&E), immunohistochemistry, or IF. For IHC staining, the sections were incubated with primary antibodies overnight at 4°C, including antibodies against GFAP (Abcam, ab7260) and OGDH (SIGMA, #B114298). Then, the peroxidase-conjugated secondary antibody (Maixin Biotechnologies, China) was incubated for 1 h at room temperature, and diaminobenzidine (DAB) substrate was used for detection. The specimens were imaged under Olympus BX50 microscope.

For IF staining, the sections were pretreated as previously described. The sections were incubated with primary antibodies overnight at 4°C, including GFAP (BIO RAD, #MCA 4684), OGDH (Sigma, #B114298), and Nestin (Cohesion Biosciences, #CQA2618). The sections were incubated with the IF-conjugated secondary antibody: goat anti-rat IgG secondary antibody (Invitrogen, #1814724) and donkey anti-mouse IgG secondary antibody (Invitrogen, #1820027) at room temperature for 1 h. The nuclei were counter-stained with DAPI (4',6-diamidino-2-phenylindole), and the images were captured using a fluorescence microscope (Olympus IX83).

For 5-hmC staining, the primary astrocytes and U87 glioma cells grown on cover slides were fixed in 4% PFA, and then permeabilized in 0.1% Triton X-100 and blocked in 1% BSA. DNA was denatured by immersing sections in 2 mol/L HCl for 30 min and then neutralized in boric acid buffer at room temperature for 10 min. After being washed with phosphate-buffered saline (PBS), the cover slides were incubated with anti-5-hmC antibody (Cell Signaling, #51660) overnight at 4°C. Donkey anti-mouse IgG secondary antibody (Invitrogen, #1820027) was used, and the images were captured using a fluorescence microscope (Olympus IX83). Fluorescence intensity was analyzed using the Olympus cellSens Dimension.

### Immunoblotting

Proteins were extracted from human glioma samples, cultured astrocytes, and glioma cells, separated by SDS-PAGE, and then transferred to PVDF membranes. The membranes were blocked in 5% milk and incubated with corresponding primary antibodies overnight at 4°C. The following primary antibodies were used: β-Actin (ABclonal, #AC026), GFAP (Abcam, #ab7260), Nestin (ABclonal, #A11861), Sox2 (ABclonal, #A19118), CD133(ABclonal, #A12711), CD44 (Cell Signaling, #3570S), IDH1^R132H^ (MXB Bio-technologies, #MAB-0733), OGDH (ABclonal, #A6391), CS (ABclonal, #A5713), SDHB (ABclonal, #A10821), FH (ABclonal, #A5688), H3K27me2 (ABclonal, #A2362), H3K27me3 (ABclonal, #A2363), H3K4me3 (ABclonal, #2357), H3 (ABclonal, #A2348), GLUD1/2 (Abcam, #ab166618), GLUL (Abcam, #ab73593), GLS (ABclonal, #A3885), and BCAT1 (Novus, #NBP2-01826). After being washed with PBST, the PVDF membrane was incubated with corresponding horse radish peroxidase-conjugated secondary antibody at room temperature for 60 min, and developed with enhanced chemiluminescence (ECL) substrate.

### Quantitative real-time PCR (qRT-PCR)

Total RNA was isolated with TRIzol according to the manufacturer’s instructions, and quantified by the Nanodrop ND-2000 spectrophotometer (ThermoFisher Scientific). cDNA was produced using Super-Script II (TaKaRa, Tokyo, Japan). qRT-PCR was performed with the SYBR Green PCR chemistry, and the OGDH qRT-PCR primers were: 5'-TTGGCTGGAAAACCCCAAAAG-3', 5'-TGTGCTTCTACCAGGGACTGT-3'.

### Metabolite analysis

For the targeted metabolite analysis, the culture media was removed, and the cells were washed with warmed PBS immediately. The metabolites were extracted using 1 mL precooled (−20°C) acetonitrile:methanol:water (2:2:1, vol/vol/vol) mixture. The cells were extracted by vortex mixing and sonicated on ice for 20 min, and protein was precipitated at −20°C for 1 h. After centrifugation at 4°C for 20 min at 14,000×g, the supernatant was collected and vacuum dried for LC–MS analysis. The metabolic analysis was performed by Shanghai Applied Protein Technology (APTBIO Shanghai, China). Amino acid analysis was performed using high-performance liquid chromatography (Shimadzu LC-20AD, Japan) coupled to quadruple time-of-flight (Q-TOF, AB Sciex 6600, USA) by the State Key Laboratory of Cancer Biology, Department of Biochemistry and Molecular Biology, The Fourth Military Medical University.

### Glutamine and BCAA determination

The *Id*h1*-wt* and *Id*h1*-mu* primary astrocytes and the *ID*H1*-WT* and *ID*H*1-MU* U87 isogenic cells were cultured with conditional DMEM indicated in the corresponding figure legends. The culture medium was collected for Gln and BCAA assays. The culture medium was collected and centrifuged at 15,000 × g for 10 min to remove cell debris and other insoluble materials. The BCAAs in the culture medium were determined using a commercial colorimetric assay kit (BioVision, K564-100). Gln in the culture medium was determined using a commercial colorimetric assay kit (Abnova, KA1627). Sample blank controls were set following the manufactory’s instructions, and optical density at 565 nm (OD_565_) was read. Microplate Spectrophotometer (ThermoFisher Scientific 1510) was used to measure the results.

### Biochemical assay

Ammonia Assay Kit (Sigma, AA0100) was used to measure ammonia concentration in the culture medium. The primary astrocytes and glioma cells were cultured in conditional DMEM with 5.5 mmol/L glucose without FBS. After 8 h, the culture medium was collected and ammonia concentration was measured. According to the manufactory’s protocol, the absorbance of each solution was measured at 340 nm (Thermo Fisher Scientific 1510). The contents of α-KG in the primary astrocytes and glioma cells cultured in 6-well plates were measured using α-KG Assay Kit (Abcam, ab83431). The concentrations of D-2HG in glioma cells were measured using D-2HG Assay Kit (Colorimetric) (Abcam, ab211070).

### Cell number determination

Cells in 96-well plates were fixed in 100 μL/well 4% PFA for cell number assessment. The fixed cells were stained by 100 μL/well 0.5% crystal violet (Sigma) in absolute ethanol for 30 min. After the stain was removed, the fixed cells were rinsed in PBS three times and dried. Crystal violet was extracted with 100 μL 10% acetic acid, and the absorbance at 590 nm was measured by a microplate spectrophotometer (Thermo Fisher Scientific 1510).

### The Cancer Genome Atlas database

Different gene expression data in mutant *IDH1/2* LGGs were downloaded from TCGA (PanCancer Atlas, 2018) and analyzed using GraphPad Software. OGDH expression and related overall survival (OS) of *IDH1/2* mutant and wild-type glioma TCGA datasets (downloaded from UCSC Xena) were analyzed using GraphPad Software. Quartiles were used as cutoff to divide samples as high- or low-expression group.

### Statistical analysis

All results are shown as mean ± SEM, and *n* values and the numbers of biological replicates for each panel in the figures were stated in the corresponding legends. Graphpad Prism (version 6.0) (Graphpad Software, La Jolla, CA, USA) was employed for statistical analysis. The normality of the data distributions was checked by the Shapiro–Wilk test. Comparisons between the two groups were performed using two-tailed unpaired Student’s *t*-test, Mann–Whitney test, and one-way ANOVA. Survival curve was analyzed using a log-rank (Mantel–Cox) test for significance. ^*^P < 0.05, ^**^P < 0.01, ^***^P < 0.001; ns, not significant.

## Supplementary Material

loae002_suppl_Supplementary_Figures_S1-S4

## Data Availability

All study data are included in the article and/or Supplementary Materials. Materials are available upon request.
