# Supplementary material for: IDH1 mutation inhibits differentiation of astrocytes and glioma cells with low oxoglutarate dehydrogenase expression by disturbing α-ketoglutarate-related metabolism and epigenetic modification
Source: Life Metab. 2024 Jan 15;3(2):loae002. doi: 10.1093/lifemeta/loae002 (PMC11749698; doi:10.1093/lifemeta/loae002)
Supplement: loae002_suppl_Supplementary_Figures_S1-S4 [file loae002_suppl_Supplementary_Figures_S1-S4.docx]

**
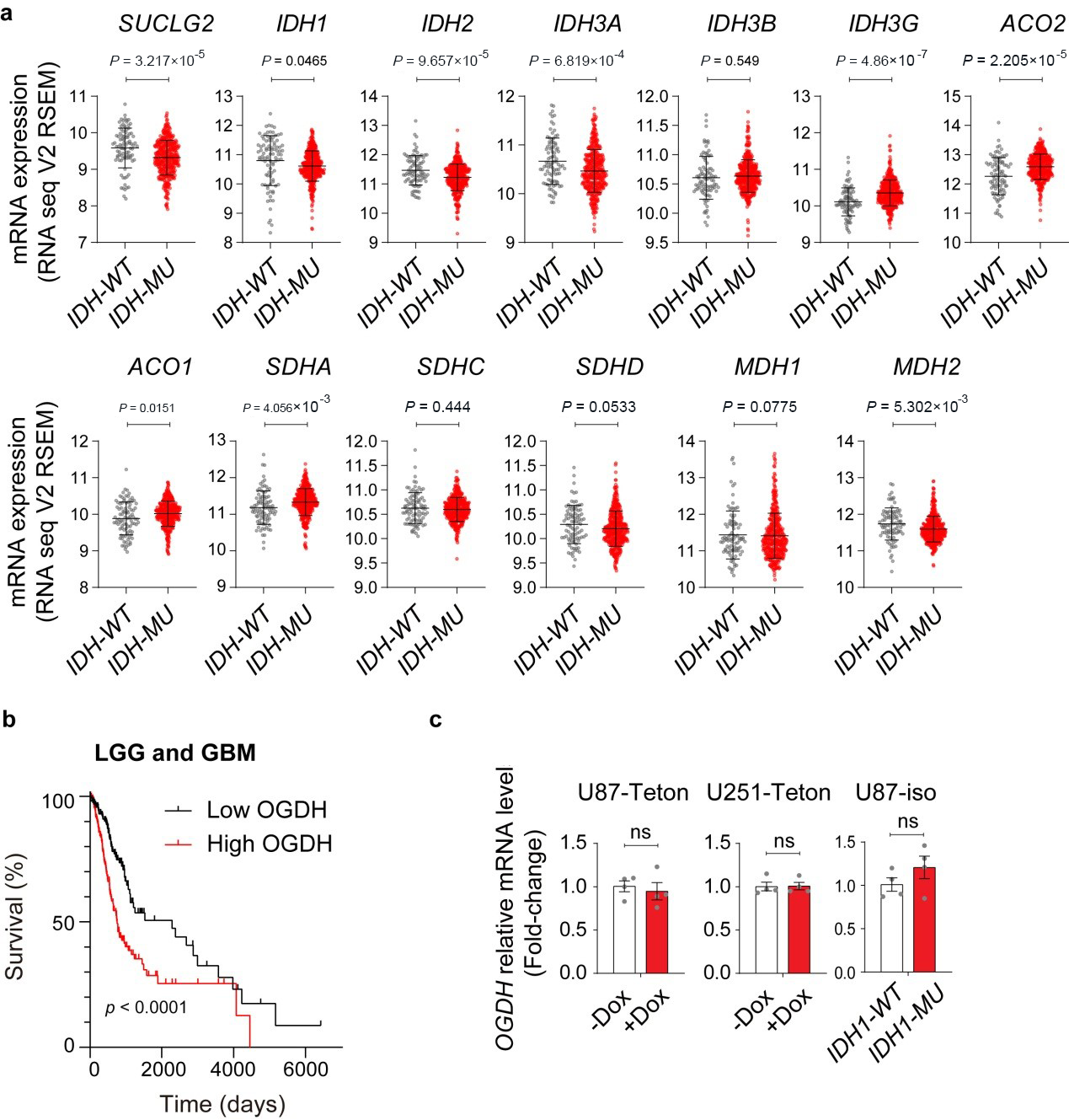
**

**Supplementary Figure S1** mRNA levels of TCA cycle enzymes in IDH-mutant gliomas. (a) The mRNA levels of TCA cycle enzymes in *IDH-wild-type* (*IDH-WT*) and *IDH-mutant* (*IDH-MU*) lower-grade gliomas (LGGs) were analyzed according to TCGA database (*n* = 92 in *IDH-WT* group, *n* = 416 in *IDH-MU* group). (b) Kaplan-Meier survival curves of the overall survival of glioma patients with low and high expression of OGDH in TCGA (LGG and glioblastoma (GBM), *n* = 172 in low OGDH group and *n* = 172 in high OGDH group). Survival curve was analyzed using a log-rank (Mantel-cox) test. (c) mRNA levels of *OGDH* in mutant IDH1-overexpressing U87 and U251 cells and *IDH1-mu* U87 isogenic cells. Data are represented as the mean ± SEM; *n* = 4. Statistical significance was determined using an unpaired Student’s  *t*-test. (^*^*P* < 0.05, ^**^*P* < 0.01, ^***^*P* < 0.001; ns, not significant).


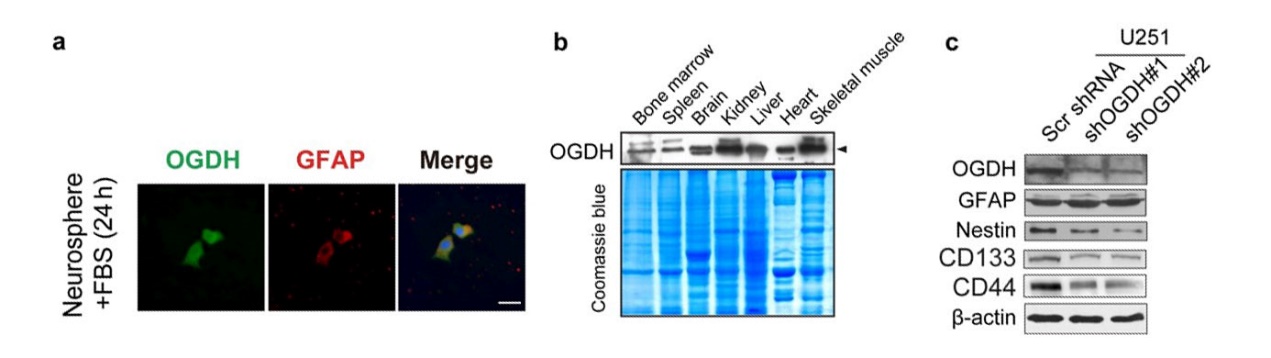


**Supplementary Figure S2** Expression of OGDH in neurospheres, different normal tissues, and glioma cells. (a) Immunofluorescence staining of OGDH (green) and GFAP (red) in astrocytes differentiated from neurospheres using FBS for 24 h, with the nuclei stained with DAPI (blue). Scale bars are 20 μm. (b) OGDH protein expression in different normal tissues. (**c**) Expression of OGDH, GFAP, Nestin, CD133, and CD44 in *IDH1-WT* U251 cells with silenced OGDH expression.


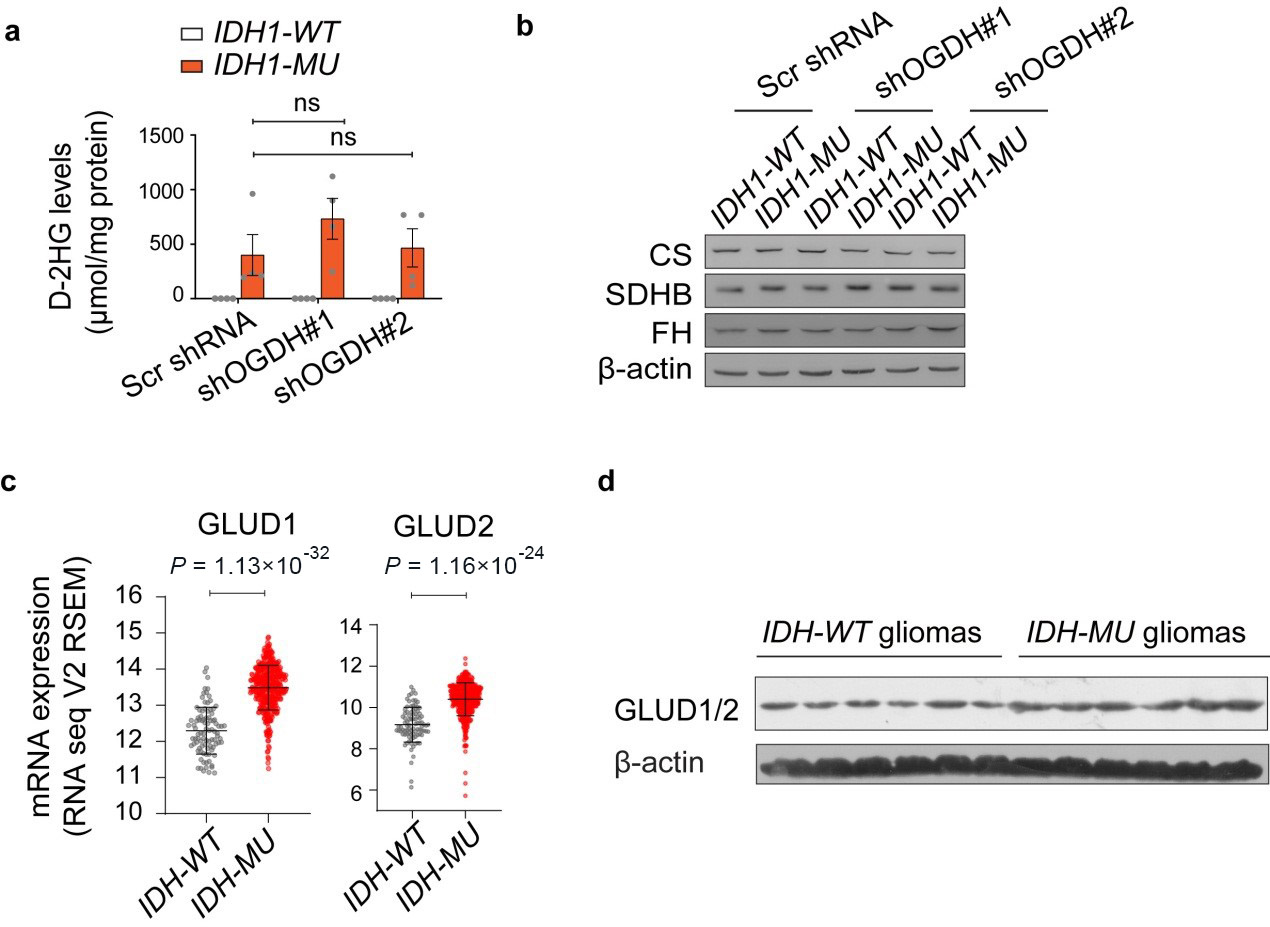


**Supplementary Figure S3** Levels of D-2HG and TCA cycle enzymes in glioma cells with silenced OGDH expression and the GLUD expression in IDH-mutated glioma samples. (a) Quantification of D-2HG contents in *IDH1-MU* (mutant) U87 isogenic cells with silenced OGDH expression. (b) Expression of CS, SDHB, and FH in U87 cells with silenced OGDH expression. (c) mRNA levels of *GLUD1/2* in *IDH-WT* and *IDH-MU* LGGs analyzed according to TCGA database. (d) Immunoblotting for GLUD1/2 in *IDH1-WT* and *IDH1-MU* glioma tissues. Data are represented as the mean ± SEM; *n* = 4. Statistical significance was determined using one-way ANOVA (a). (^*^*P* < 0.05, ^**^*P* < 0.01, ^***^*P* < 0.001; ns, not significant).


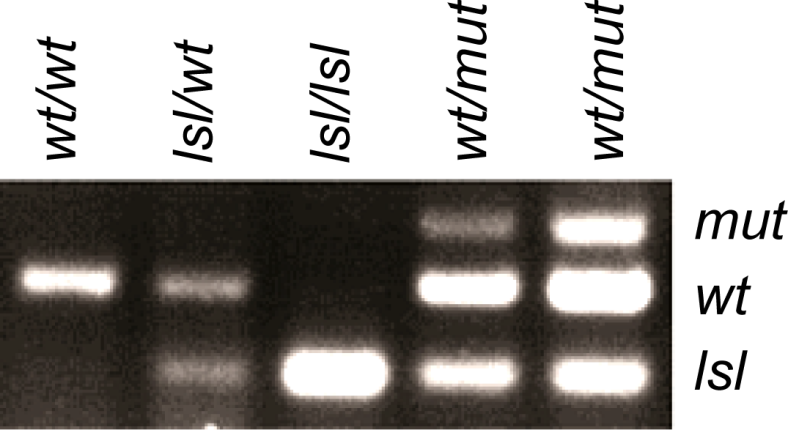


**Supplementary Data Figure S4** *Idh1^lsl-R132H/+^*;*GFAP-Cre* (*Idh1-mu*), and *Idh1^+/+^*;*GFAP-Cre* (*Idh1-wt*) mice genotyping. Sense and antisense primers were 5'-ACCAGCACCTCCCAACTTGTAT-3', 5'-AGGTTAGCTCTTGCCGATCCGT-3', and 5'-CAGCAGCCTCTGTTCCACATAC-3'. The predicted PCR product sizes of mutant-type, wild-type and *lsl* are 394 base pairs (bp), 307 bp, and 223 bp, respectively.
